# Canadian Association of Gastroenterology Position Statement: Use of Cannabis in Gastroenterological and Hepatic Disorders

**DOI:** 10.1093/jcag/gwy064

**Published:** 2018-11-08

**Authors:** Christopher N Andrews, Shane M Devlin, Bernard Le Foll, Benedikt Fischer, Frances Tse, Martin Storr, Stephen E Congly

**Affiliations:** 1 Division of Gastroenterology and Hepatology, Cumming School of Medicine, University of Calgary, Calgary, Alberta, Canada; 2 Translational Addiction Research Laboratory, Campbell Family Mental Health Research Institute, Centre for Addiction and Mental Health, Toronto, Ontario, Canada; 3 Acute Care Program, Centre for Addiction and Mental Health, Toronto, Ontario, Canada; 4 Departments of Family and Community Medicine, Pharmacology and Toxicology, Psychiatry, Institute of Medical Science, University of Toronto, Toronto, Ontario, Canada; 5 Campbell Family Mental Health Research Institute, Centre for Addiction and Mental Health, Toronto, Ontario, Canada; 6 Institute for Mental Health Policy Research, Centre for Addiction and Mental Health, Toronto, Ontario, Canada; 7 Department of Gastroenterology and Hepatology, McMaster University, Hamilton, Ontario, Canada; 8 Department of Medicine, University of Munich and Center of Endoscopy, Starnberg, Germany

**Keywords:** Cannabis, Hepatic disorders, Gastroenterological disorders, Position statement

Medical cannabinoid products are widely used in Canada to treat medical symptoms of all kinds, and gastrointestinal (GI) symptoms are among the most commonly cited reasons for use (1). Cannabis is also widely used recreationally (2), and legalization of recreational use has occurred in Canada.

Currently, cannabis is not an approved therapeutic product in Canada. However, health care practitioners may authorize the use of cannabis in various forms or synthetic cannabinoids for the relief of symptoms associated with a variety of disorders which have not responded to conventional medical treatments. These include pain and spasticity due to multiple sclerosis; severe nausea and vomiting related to cancer chemotherapy; loss of appetite and body weight in cancer patients and patients with HIV/AIDS; chronic noncancer pain (mainly neuropathic); severe refractory cancer-associated pain; insomnia and depressed mood associated with chronic diseases (HIV/AIDS, chronic noncancer pain); and symptoms encountered in the palliative/end-of-life setting (3). Yet, evidence supporting the safety and efficacy of cannabis for the treatment of many of these conditions is often limited and inconsistent.

Certainly, a biological rationale exists for cannabinoids to have possible benefit for GI symptoms based on its known physiologic actions (3, 4) but research in the field has been hampered by its historical illegality, plus the inability to patent a natural product which is widely available. However, there are also concerns regarding potential adverse effects of acute and chronic cannabis use including dependence, tolerance, psychiatric disorders, poor school or work performance, nervous system disorders, vascular and cardiac disorders, carcinogenesis and gastrointestinal disorders such as cannabis hyperemesis syndrome (CHS) and fibrosis progression in chronic hepatitis C.

The pharmacodynamics and pharmacokinetics of inhaled and ingested cannabis are reasonably well known and are reviewed elsewhere (3). However, very few randomized clinical trials of cannabis exist for GI indications, and the evidence that does exist is typically low or very low quality (such as case series or open-label studies). Further hampering a clear picture is the lack of standardization of cannabinoid products, which may contain hundreds of bioactive compounds, of which most have unknown effects. Two cannabinoids are better understood and are typically used to guide prescribing (specifically delta-9 tetrahydrocannabidiol [THC] and cannabidiol [CBD]), but these also vary substantially across strains of cannabis. Variability in inhalation or ingestion methods can also dramatically affect levels and effects of bioactive substances in the body.

Given these factors, the Canadian Association of Gastroenterology (CAG) has determined that guidance is required on the issues of relevance for clinical practitioners in the area of cannabis benefits and harms and particularly so with the anticipation of increased recreational use in Canada and more widespread medical use worldwide.

## METHODS

The lack of prospective clinical trial data for GI indications of cannabis precludes the possibility of meta-analysis in most cases. It was anticipated that the quality of evidence would generally be low or very low, and for some statements, only indirect evidence would exist. Where appropriate, a nonexhaustive evidence review was undertaken (MEDLINE, full text only, English language, humans only, with keywords ‘cannab*’ and ‘marijuana or hemp’ with the disease state) to inform the statements. Literature of significant importance is cited, but readers will be directed to appropriate recent reviews for more comprehensive information. It should also be noted that the CAG position statement is based on the literature as it stands at this time and could be subject to change in the future. As the literature grows in the field of cannabis and GI, especially if and when new clinical trial data become available, it is possible that the recommendations may change. The position statements were reviewed and approved by the CAG Practice Affairs committee.

### General Statements

#### Statement

Medical cannabis use should not replace Health Canada–approved medical therapy for treatment of any gastroenterologic or hepatologic disease if the approved therapy is available and has not been used.

#### Commentary

Pharmaceutical agents approved by Health Canada have been evaluated to very high standards with both preclinical testing and prospective trials in order to obtain an indication for use. Cannabinoid medications have a very limited scope of approved GI-related indications at this time: treatment of severe nausea and vomiting associated with cancer chemotherapy (dronabinol, nabilone) (5, 6), AIDS-related anorexia associated with weight loss (dronabinol) (5), and intractable pain related to advanced cancer (nabiximols) (7). However, medical cannabis (as dried flower for inhalation, oil or other oral forms) is not an approved therapeutic substance per Health Canada, but there is a variety of conditions for which health care practitioners can authorize use (not limited in scope as long as a practitioner justifies its use in a medical document) (3).

The difference between treating a disease and managing the symptoms of a disease must be stated because patients and much of the lay literature available to the public do not necessarily distinguish between these aspects. Since the evidence for disease-modifying treatment with cannabis is scant to nonexistent for most indications, we recommend using recognized and approved therapies over cannabis. This is especially important in diseases where the risk of significant morbidity may occur with untreated or poorly treated disease (e.g., viral hepatitis, autoimmune hepatitis, inflammatory bowel disease, GI malignancies) and for which effective and approved treatments exist. Concurrent use of cannabis for symptom control alongside approved therapies may be reasonable, provided the risk of harm appears low.

#### Statement

If patients decide to use cannabis recreationally or for a medical reason, they should only use product from a Canadian licensed producer (LP) to ensure quality, lack of contamination, and reliable potency information on the product.

#### Commentary

Cannabis grown outside of licensed production facilities is typically not tested for contaminants, which may include fungi, pesticides or other adulterants (8). This is of particular concern if a patient is immunosuppressed. Dried flower or cannabis oil from LPs also has accurate information on potency of THC and CBD, which is essential for medical dosing. Finally, unregulated cannabis products (with the exception of authorized home production) are illegal.

### Inflammatory Bowel Disease

#### Statement

Cannabis does not appear to alter the course of disease in IBD (for better or worse) based on current evidence.

#### Commentary

Cannabis has long been described in terms of its ability to ameliorate a variety of gastrointestinal symptoms including anorexia, nausea, abdominal pain and diarrhea (9). These properties could potentially make it an attractive treatment for the common gastrointestinal symptoms associated with inflammatory bowel disease (IBD).

Cannabis as a potential therapeutic option that could target the aberrant gastrointestinal immune response associated with IBD is not without biologic plausibility. The gastrointestinal tract is replete with endocannabinoid receptors that have been demonstrated to participate in the regulation of motility and maintenance of the epithelial barrier (10, 11). Moreover, amelioration of inflammation in a murine ileitis model has been demonstrated when the endogenous cannabinoid 2 receptor (CB2R) was stimulated with a receptor specific ligand (11). Cannabidiol (CBD) is a key component of cannabis that lacks psychotropic properties but is likely responsible for many of the gastrointestinal effects of cannabis. It has recently been demonstrated in vitro to reduce inflammatory cytokine expression from human colonic cells of patients with IBD (12).

Data in humans with IBD have largely focused, until recently, on observational studies evaluating the demography of patients and the rationale for the use of cannabis in this patient population (13–17). In one prospective study, 12% of patients with IBD were active users of cannabis, and 39% were past users. Of the users, 16% used for symptom control, the majority of whom reported a subjective benefit (16). In a cross-sectional study of Canadian IBD patients who used cannabis for symptom control, users were statistically more likely to have a history of chronic analgesic utilization, use other complementary therapies and have a history of abdominal surgery (15). Similarly, the self-reported use of cannabis was found to be associated with a history of surgical resection for Crohn’s disease (CD) in a Canadian study (17). The association with abdominal surgery must be interpreted cautiously because it may reflect a desire for symptom control in a cohort of patients with more disabling symptoms related to a potentially more aggressive disease course rather than implying causality. Nevertheless, in the majority of studies, a significant proportion of patients subjectively felt that cannabis use was associated with improvements in symptom control. Moreover, users in the study by Lal et al. were more likely than nonusers to be interested in participating in a clinical trial evaluating cannabis use for the treatment of IBD (15).

Perhaps the most important question facing gastroenterologists is whether there is evidence to support the use of cannabis or cannabinoid as a therapeutic agent that influences the inflammatory disease activity, rather than just symptoms, in patients with IBD. This is an arena in which there is a relative paucity of data to guide clinicians, and a comprehensive overview is beyond the scope of this review.

The first study evaluating this question was a retrospective Israeli study, which reported the clinical symptoms and need for surgery in 30 patients with CD who used inhaled cannabis. The disease activity, as measured by a Harvey Bradshaw Index (HBI), was lower in patients who used cannabis, but no objective measures were otherwise recorded (18). The same group then conducted an eight-week prospective, placebo-controlled trial in 21 medically refractory CD patients comparing the use of inhaled cannabis cigarettes containing 115 mg of tetrahydrocannabinol (THC) with cigarettes from which THC had been extracted. At the end of the study, significantly more patients treated with THC achieved a 100-point reduction in the Crohn’s disease activity index (CDAI) than those treated with THC-free cigarettes (19). The only objective measure of disease activity that was assessed was C-reactive protein which did not change over the study duration in either treatment group. The same group evaluated CBD alone as a therapeutic agent in a placebo-controlled, randomized controlled trial (RCT) in 20 patients with medically refractory CD and found that no change in the CDAI was observed over an eight-week period (20). All three of these studies were supported by an Israeli licensed producer of cannabis.

A multi-centre, double-blind randomized controlled trial from the United Kingdom evaluated 60 patients with mild to moderate ulcerative colitis who were treated with a CBD-rich botanical extract or placebo over a 10 week period. The primary endpoint was the proportion of patients in remission at the end of the study. A per-protocol analysis was required due to a higher proportion of protocol violations in the CBD-treated patients due to higher rates of non-adherence and adverse events in this group. By intention to treat analysis, the rates of clinical remission were not different between the two groups. In the per-protocol analysis, there was a statistically significant reduction in the partial Mayo score in the CBD treated group. However, the reduction in fecal calprotectin between the two groups did not differ by study end (21).

The most recent study was published in 2018 in abstract form only (22). The study was a randomized controlled trial comparing inhaled cigarettes containing 11.5 mg of THC with placebo (THC-free) cigarettes in 28 patients with medically refractory moderate UC. The primary endpoints included both clinical disease activity indices and endoscopic disease activity. Both disease activity and the endoscopic disease severity decreased significantly in the THC-treated group compared with the placebo-treated group after eight weeks of therapy. However, it is worth noting the baseline CRP and fecal calprotectin levels were numerically higher in the placebo-treated groups than in the treatment group. Furthermore, the quality of the study cannot be fully ascertained until published in full.

At present, evidence that use of cannabis is associated with objective measures of reduction of inflammation is largely lacking and limited to small studies with insufficient power to detect a clinically relevant difference. Large, randomized, placebo-controlled trials assessing objective outcome measures using standardized preparations of cannabis with long-term follow-up of safety and effectiveness are therefore needed. There is insufficient evidence to recommend the use of cannabis as an anti-inflammatory treatment option for IBD at this time.

### Hepatology

#### Statement

Although cannabinoids have been associated with improved outcomes in nonalcoholic fatty liver disease and alcoholic fatty liver disease in epidemiological studies, there is insufficient data to support their use in these diseases.

#### Commentary

Currently, there is limited information regarding the use of cannabis in liver disease. The majority of studies are cohort studies with only one small RCT to date; the literature in this field was recently reviewed by Goyal et al. (23).

Endocannabinoids are thought to affect the liver in several ways. First, they are associated with fat accumulation through increased lipogenesis throughout the body and decreased rates of lipolysis (24), further augmented by appetite stimulation. Secondly, cannabinoids are thought to act on the CB1 and CB2 receptors. The CB1 receptors are thought to be profibrotic (25) and associated with the development of nonalcoholic fatty liver disease (NAFLD) (26) and steatosis (27). On the other hand, CB2 receptors, which are upregulated in chronic liver disease (28), are thought to be protective against fibrosis but can increase steatosis (29).

### Alcoholic Liver Disease (ALD)

The use of cannabinoids with ALD may have a possible protective effect. In a mouse model, cannabinoids reduced inflammation, oxidative stress and steatosis in ALD (30). Current human data are based on analysis of the 2014 National Inpatient Sample (NIS) of patients with a history of past/current abusive alcohol use based on ICD-9 codes. In this cohort study, cannabis use was associated with decreased rates of alcoholic steatosis, hepatitis, cirrhosis and hepatocellular carcinoma (31). A major limitation of this study is that it is an administrative study reliant on correct coding for steatosis/hepatitis and cannabis use by ICD-9 codes, which may not be completely accurate. Further study in this area is needed.

### Nonalcoholic Fatty Liver Disease (NAFLD)

At this time, the only human studies looking at NAFLD are cross-sectional in nature using two American datasets: the NIS (32) and the National Health and Nutrition Examination Survey (NHANES) (33). In both studies, cannabis use was associated with a lower prevalence of NAFLD with odds ratios ranging between 0.49–0.68, with a potential dose-related effect (32). These studies are limited given that the diagnosis of NAFLD was made by either ICD-9 codes or abnormal liver tests with steatosis on ultrasound. Further, potential mechanisms of this effect have not been well established, and so further research is needed before a recommendation can be made on cannabis’s potential use in NAFLD.

#### Statement

Cannabis likely increases hepatic fibrosis and steatosis in patients mono-infected with hepatitis C, and so its use is not recommended.

#### Commentary

At this point, there is no human data regarding the role of cannabis with chronic hepatitis B, but in vitro cannabis has no effect on the virus (34). The effect of cannabis in patients in chronic hepatitis C has been variable although in vitro; cannabinoids may have an antiviral effect on hepatitis C (34). Cannabis use in chronic hepatitis C has been associated with moderate to severe fibrosis (35), more rapid fibrosis progression (36) in two cohorts and higher rates of steatosis (37). Conversely, a more recent Canadian study found cannabis had no effect on steatosis nor fibrosis (38).

Historically, cannabis was used as an adjunct agent in hepatitis C treated with interferon and ribavirin. Cannabis use was linked with higher rates of treatment completion and cure (39, 40). With the availability of extremely potent direct acting antiviral agents with minimal side effects, adjunct use of cannabis is not recommended.

In patients coinfected with hepatitis C and HIV, cannabis use was been found to reduce the rate of steatosis in a French cohort of 838 patients (41). Furthermore, this cohort suggests that cannabis can reduce the risk of insulin resistance in coinfected patients (42). The negative impact of cannabis on fibrosis seems to be attenuated in co-infected patients, with two North American cohort studies showing no association of smoking cannabis with advanced fibrosis (28, 43). It should be noted that the Canadian series showed a possible signal of increased cannabis use being associated with higher risks of fibrosis progression (43). The different effects of cannabis between the co-infected groups and mono-infected groups is unclear, and further study is warranted.

#### Statement

Cannabis or synthetic analogues are possibly hepatotoxic.

#### Commentary

Cannabis has been associated with possible hepatotoxicity, with one small series showing an association with mildly abnormal ALT, AST and ALP levels (35–54%), hepatomegaly (57.7%) and splenomegaly (73.1%), although other etiologies of liver disease were not assessed in this study (44). There have been four case reports of liver failure associated with the use of either cannabis or synthetic analogues (45–48), but two of the cases had other more likely etiologies than cannabis or an analogue (45, 46).

#### Statement

Cannabis use is not an absolute contraindication to liver transplantation, but its use should be assessed on a case by case basis; in the context of other addictions, cannabis use is not recommended.

#### Commentary

The use of cannabis in the context of liver transplantation remains controversial, with a minority of North American programs transplanting patients who use cannabis (49). Concerns of its use include possible impact on graft survival, medication compliance and addictions (50). However, cannabis use does not seem to have an adverse impact on liver transplant waitlist survival in two retrospective case series from Michigan (51) and San Francisco (52).

We would suggest that although cannabis use is not an absolute contraindication to transplantation, its use should be assessed on a case by case basis. We would discourage its use recreationally, and the risks and benefits would need to be considered in the context of medical use. If individuals have a history of addictions including street drugs or alcohol, we would recommend abstinence from cannabis and completion of any recommended treatment program by an addictions specialist.

In summary, the impact of cannabis on liver disease remains unclear and seems to have variable effect depending on the underlying disease. Potentially, cannabinoids may have a future role in the management of NAFLD and ALD, but more studies are required. Given the totality of evidence, its use in patients with hepatitis C is not recommended. Cannabis by itself should not be an absolute contraindication to transplant assessment but should be assessed on a case by case basis; in the context of other addictions, its use would not be recommended.

### GI Symptom Control

#### Statement

Cannabis and cannabinoids may be helpful for GI symptom control, especially short-term use for refractory nausea, vomiting, diarrhea or abdominal pain where conventional therapies have failed.

#### Commentary

This concept is based on the approved indications for cannabis (5, 6) and its mechanism of action (4, 53). A meta-analysis of randomized controlled trials of cannabinoid use for symptom control (54) found moderate-quality evidence to support the use of cannabinoids for the treatment of chronic pain and low-quality evidence suggesting that cannabinoids were associated with improvements in nausea and vomiting due to chemotherapy and weight gain in HIV infection. However, efficacy in non-GI related chronic pain disorders does not necessarily equate to efficacy in functional GI disorders, such as irritable bowel syndrome or functional dyspepsia. The vast majority of these trials used oral formulations of cannabinoids, and adverse effects (typically mild) related to cannabis were common. Clinical trials for other GI disorders are awaited, and long-term effectiveness and safety of cannabis for chronic gut symptoms (i.e., functional gut disorders) remain unknown. Thus, cannabis or cannabinoids should not be considered first-line treatment for nausea, vomiting unrelated to chemotherapy, or any other off-label GI symptom.

### Cannabis hyperemesis syndrome

#### Statement

Cessation of cannabis or cannabinoid use is recommended as the mainstay of treatment for cannabis hyperemesis syndrome (CHS).

#### Commentary

Cannabis hyperemesis syndrome is a recently recognized disorder of cyclic vomiting associated with chronic cannabis use (55). Other features of CHS may include morning nausea, abdominal pain or frequent hot showers or baths for symptom relief. Patients typically have a history of daily cannabis use, often for years, before the onset of symptoms. Use was reported as inhaled in almost all reports, with no reports of CHS in patients with exclusive oral ingestion of (nonsynthetic) cannabinoids. Although population-based incidence data are not available, patients commonly present to emergency departments and GI practices as one of the most frequent causes of chronic nausea and vomiting in younger people (56) (C. N. Andrews, personal communication, October 2018). The cause is unknown at this time, although hypotheses suggest the cause may be multifactorial; altered cannabinoid metabolism, complex pharmacodynamics at the CB-1 receptor and genetic variability are all proposed to play a role.

Small case series have suggested a possible benefit for intravenous haloperidol and topical capsaicin cream for acute exacerbations of CHS, with lesser apparent effectiveness of standard anti-emetic therapy, but no controlled trials have been performed (55, 57). Cessation of cannabis use appears to be the best treatment, although the quality of evidence is similarly very low (55). However, cessation has the advantage of long-term resolution of symptoms in some cases. Many patients may have a co-existing cannabis use disorder, which may limit their ability to stop using it (58).

### Risk Reduction

#### Statement

Recently published Lower-Risk Cannabis Use Guidelines (LRCUG) (59) systematically reviewed available evidence and made 10 major recommendations for lower-risk use: (1) the most effective way to avoid cannabis use-related health risks is abstinence; (2) avoid early age initiation of cannabis use; (3) choose low-potency tetrahydrocannabinol (THC) or balanced THC-to-cannabidiol (CBD) ratio cannabis products; (4) abstain from using synthetic cannabinoids; (5) avoid combusted cannabis inhalation and give preference to nonsmoking use methods; (6) avoid deep or other risky inhalation practices; (7) avoid high-frequency (e.g., daily or near-daily) cannabis use; (8) abstain from cannabis-impaired driving; (9) populations at higher risk for cannabis use-related health problems should avoid use altogether; and (10) avoid combining previously mentioned risk behaviors (e.g., early initiation and high-frequency use).

#### Commentary

The LRCUG would generally apply to recreational use, while recognizing that medical use for GI disorders may require or result in noncompliance with select recommendation details (e.g., use frequency, administration method) in the short term. Specific evidence-based medical benefits from medical cannabinoid use may supersede general risk management considerations on a case-by-case basis. Otherwise, risk reduction concerns should generally outweigh perceived benefit where there is a lack of evidence. Any licensed physician in Canada may authorize the use of cannabis for medical purposes for a patient; therefore, the CAG recommends physicians familiarize themselves with important aspects to consider before authorizing a patient for medical use. Moreover, with recreational use being so common, it also behooves physicians to understand the risks involved for patients and to be able to counsel them accordingly.
